# Intravitreal Bevacizumab Injection Attenuates Diabetic Retinopathy in Adult Rats with Experimentally Induced Diabetes in the Early Stage

**DOI:** 10.1155/2018/9216791

**Published:** 2018-05-09

**Authors:** Jiao Lv, Miao-Miao Chen, Zhi-Hao Mu, Fang Wang, Zhong-Yi Qian, Lei Zhou, Qiu-Ting Guo, Zhi-Min Zhao, Yu-Ping Pan, Xin-Yu Liao, Zhi-Hong Yang, Ning Cai, Shu-De Li, Ying-Ying Zou

**Affiliations:** ^1^Department of Pathology and Pathophysiology, Faculty of Basic Medical Sciences, Kunming Medical University, 1168 West Chunrong Road, Kunming 650500, China; ^2^Department of Ophthalmology, First Affiliated Hospital of Kunming Medical University, 295 Xi Chang Road, Kunming 650032, China; ^3^Department of Morphological Laboratory, Faculty of Basic Medical Sciences, Kunming Medical University, 1168 West Chunrong Road, Kunming 650500, China; ^4^The Key Laboratory of Stem Cell and Regenerative Medicine of Yunnan Province, Kunming Medical University, 1168 West Chunrong Road, Kunming 650500, China; ^5^Department of Biochemistry and Molecular Biology, Faculty of Basic Medical Sciences, Kunming Medical University, 1168 West Chunrong Road, Kunming 650500, China

## Abstract

Diabetic retinopathy is the leading cause of blindness, yet its treatment is very limited. Anti-VEGF drug has been widely applied in ocular disease, but its effects on diabetic retinopathy and the underlying mechanism have remained to be fully explored. To elucidate the role of anti-VEGF treatment, we sought to determine the effects of bevacizumab on diabetic neurovascular changes extending from the 3rd to 9th week with induced diabetes in adult rats. The retinal neurovascular changes included increased expression of VEGF, nNOS, iNOS, eNOS, and NO in the course of diabetes progression. In diabetic rats given bevacizumab injection, the ganglion cell loss and alterations of retinal thickness were ameliorated. In this connection, the immunofluorescence labeling of the above biomarkers was noticeably decreased. Along with this, Western blotting confirmed that bevacizumab treatment was associated with a decrease of VEGF, Flk-1, and cAMP response element binding and protein kinase C protein expression. The present results suggest that bevacizumab treatment in the early stage of the retinopathy may ameliorate the lesions of retinopathy, in which VEGF/Flk-1 signaling has been shown here to play an important role.

## 1. Introduction

Diabetic retinopathy (DR) is the commonest complication of diabetes and the leading cause of blindness in developed countries [1–3]. Although laser therapy and vitreoretinal surgery have been widely adopted, their effectiveness is limited and they mainly target at the later stages of the disease [1]. The control of the risk factors for diabetes in the early stage is therefore desirable to find new ways to identify, to prevent, and, hopefully, to eventually cure the diabetic retinopathy [2, 4].

The retina is a highly vascularized neural tissue [4]. Traditional treatments have focused on pathological changes of vasculature and intervention in the later stages. Recent studies have shifted the focus to the role of nonvascular cells, such as neurons, astrocytes, and Müller cells, in the progression of DR and accompanying molecular changes [5]. Further understanding of the biology of DR, especially the relationship between vascular and neural changes, would contribute to the development of novel treatments [4].

Vascular endothelial growth factor (VEGF) has been reported to play a pivotal role in the development of DR [3, 6, 7]. VEGF levels are increased in animals with experimental diabetes and patients with diabetic retinopathy [3]. Previous studies have reported that inhibition of VEGF with anti-VEGF drugs could reduce retinal permeability and neovascularization [8, 9]. These results have resulted in the development of anti-VEGF strategies such as bevacizumab as potential new therapies [10, 11]. Bevacizumab has been shown to ameliorate rapid regression of retinal neovascularization and improve visual acuity and decrease retinal thickness [3, 12]. There is ample evidence supporting its effect on preventing retinal angiogenesis in DR [13]. Though many clinical studies have confirmed its efficacy [14–16], it remains to be fully explored how bevacizumab might affect both neuronal and vascular changes and contributes to functional improvement. Treating DR using bevacizumab at a late stage while lesion is severe is often unsatisfactory.

cAMP response element binding (CREB), a cellular transcription factor important to angiogenesis, could be activated by VEGF through phosphorylation of VEGF receptor 2 (Flk-1) tyrosine kinase. Protein kinase C (PKC) is an important mediator of CREB phosphorylation [17]. In light of this, we hypothesized that CREB-induced angiogenesis might play an important role in DR.

This study was therefore aimed to ascertain whether bevacizumab may be applied in the early stage to improve its efficacy. Meanwhile, we also aimed to determine on how bevacizumab might influence the neuronal and vascular changes in the early stage of DR. The efficacy of bevacizumab on these changes, including some signaling pathways, is therefore explored.

In consideration of the above, we have carried out this study in two phases. Firstly, the neuronal and vascular changes during the development of diabetes were examined. This was to determine the critical stage for intravitreal bevacizumab injection. Secondly, we sought to determine how bevacizumab treatment might reduce retinal pathological damage in the early stage of diabetes.

## 2. Material and Methods

### 2.1. Induction of Diabetes

Animal experiment protocols were performed in accordance with the National Institutes of Health Guide for the Care and Use of Laboratory Animals (NIH publication number 80-23) revised 1996. One hundred and twenty adult male Sprague Dawley rats (Dashuo, Chengdu, China), weighing 300–340 g, were divided into 8 groups according to duration of diabetes mellitus (DM) since its induction, including normal control (NC and NC11w) group (*n* = 12), DM3w, DM6w, DM9w, and DM11w groups (*n* = 16), and different treatments, including diabetes with a single intravitreal injection of sodium lactate Ringer's (DM11w + LR) group (*n* = 16) and diabetes with a single intravitreal injection of bevacizumab (DM11w + B) group (*n* = 16).

Diabetes was induced by a bolus intraperitoneal injection of streptozotocin (STZ, 65 mg/kg in citrate buffer; Sigma, St. Louis, MO, USA), while the normal control group received the same volume of citrate buffer. The body weight was recorded, likewise, for the fasting blood glucose level at 4 days after STZ injection. Diabetes was defined by a fasting blood glucose concentration of over 16.67 mmol/L. In our previous study, we have monitored the blood glucose level and found it maintained at a high level [18].

### 2.2. Intravitreal Injection

Intravitreal injection was administered at 6 weeks after diabetes induction. Rats were anaesthetized by an intraperitoneal injection of 10% chloral hydrate (0.35 ± 0.05 mL/100 g, Hengtai Chemical Co. Ltd., Jiangsu, China). The pupil was dilated by Tropicamide (Bausch & Lomb Freda Pharmaceutical Co. Ltd., Shandong, China) topically anesthetized by Benoxil (Santen Pharmaceutical Co. Ltd., Osaka, Japan). The injection site was located temporal at 3 o'clock of corneal limbal and 1.0–1.5 mm to the limbus. Firstly, we used a NovoPen (Novo Nordisk Pharmaceutical Co. Ltd., Denmark) to pierce into the vitreous cavity. Next, 3 *μ*L of sodium lactate Ringer's or bevacizumab (25 mg/mL, Roche Pharmaceutical Co. Ltd., Schweiz) was injected into the vitreous body with a microsyringe (YuanQuan Biological Technology Co. Ltd., Shanghai, China). After the needle was withdrawn, pressure was applied at the injection site with a cotton swab, and Chloramphenicol Eye Drop (Medco Huakang Pharmaceutical Co. Ltd., Sichuan, China) was applied for the following three days. Rats with any postoperative complications, for example, cataract, were excluded from the study.

### 2.3. Paraffin Embedding and Sectioning

For histology and immunohistochemistry and double immunofluorescence labeling, four rats in each group were anesthetized with 10% chloral hydrate (4 mL/kg body weight) and perfused with filtered saline (150 mL and 12 mL/min), followed by 4% paraformaldehyde in phosphate-buffered saline (PBS), pH 7.4 (500 mL and 12 mL/min). The eyeballs were removed, dehydrated, embedded in paraffin, and cut into 4 *μ*m thick sections. The sections were transferred to silane-coated microscope slides and dewaxed.

### 2.4. H&E Staining

After dewaxing and hydration, the paraffin sections were processed for routine hematoxylin and eosin (H&E) staining.

### 2.5. Double Immunofluorescence Labeling

Normal control rats, rats of the diabetes groups (DM3, 6, and 9w), and different treatments groups (DM11w + LR and DM11w + B) were used for immunofluorescence studies. Paraffin coronal sections of the eyeball derived from different time points were incubated in primary antibodies including rat-specific vascular endothelial growth factor (VEGF, 1 : 150, Santa Cruz Biotechnology, Dallas, TX), rat-specific Flk-1 (1 : 150, Santa Cruz Biotechnology, Dallas, TX), rabbit-specific cAMP-response element binding protein (CREB, 1 : 150, Millipore, Darmstadt, Germany), and rat-specific PKC (1 : 150, Abcam, Cambridge, UK). NeuN (1 : 250, Abcam/Millipore) was used for neuronal labeling and GFAP (1 : 500, Abcam) for astrocyte labeling. After incubation and washing, FITC-conjugated and Cy3-conjugated secondary antibodies were added. After mounting, images representing at least one eyeball section each from four rats at different time points were captured under a confocal fluorescence microscope (Olympus, Tokyo, Japan). Immunofluorescence labeling for the various antibodies directed against the respective cell types was consistent and reproducible across different rats.

Semiquantification of CREB and Flk-1 by immunofluorescence was conducted with ImageJ (version 1.50i software; ImageJ, National Institutes of Health, Bethesda, MD, USA). Immunofluorescent images were transformed into 16-bit grayscale images. Areas with immunofluorescence expression were selected, and integrated density was measured. The background immunofluorescence intensity was also measured in an area without immunofluorescence. The data of corrected integrated density was gained by subtracting the background intensity from the integrated density.

### 2.6. Western Blot

Fresh tissue samples were collected from the retina. To analyze the expression levels of respective proteins, equal amounts of total proteins were subjected to 10% (*w/v*) SDS-PAGE and transferred onto polyvinylidene fluoride (PVDF) membranes (Millipore, Darmstadt, Germany). Membranes were then blocked with 5% skim milk for 2 hours at room temperature and probed with anti-VEGF (1 : 500; Santa Cruz Biotechnology, Dallas, TX), anti-Flk-1 (1 : 1000; Santa Cruz Biotechnology, Dallas, TX), anti-CREB (1 : 1000; Millipore, Darmstadt, Germany), anti-PKC (1 : 750; Abcam, Cambridge, UK), anti-*β* actin (1 : 1000; Santa Cruz Biotechnology, Dallas, TX), anti-GAPDH (Abcam, Cambridge, UK), anti-nNOS (1 : 1000, BD Biosciences, USA), anti-iNOS (1 : 750, Santa Cruz Biotechnology, Dallas, TX), and anti-eNOS (1 : 750, Santa Cruz Biotechnology, Dallas, TX) antibodies at 4°C overnight. Subsequently, membranes were incubated with horseradish peroxidase- (HRP-) conjugated goat anti-rabbit (Thermo Fisher Scientific, Waltham, MA) or rabbit anti-rat (Thermo Fisher Scientific, Waltham, MA) IgG for 2 hours at room temperature after three times TBST washing and then reacted with a prolight HRP agent (Santa Cruz Biotechnology, Dallas, TX). The result of chemiluminescence was recorded with an imaging system and semiquantified using the ImageJ software (National Institutes of Health, Bethesda, MD).

### 2.7. Nitrite Assay

The total amount of NO (mM) in the retina was assessed with a colorimetric assay kit (US Biological, Swampscott, MA) by the Griess reaction, detecting nitrite a stable reaction product of NO and NO^2−^. Briefly, 85 *μ*L of each sample was added to 5 *μ*L of nitrate reductase followed by 5 *μ*L of enzyme cofactor according to the manufacturer's instructions and incubated for 1 h at room temperature. Five microliters of enhancer was then added and incubated for 10 min. Following this, 50 *μ*L of Griess reagent R1 and R2 was added to the above solution and the color could develop at room temperature for 10 min. The optical density of the samples was measured at 540 nm using the GENios microplate reader (Tecan, Austria GmbH, Salzburg, Austria). Nitrite concentration was determined from a nitrite standard curve.

### 2.8. Retinal Thickness Assay and Ganglion Cell Counting

The sections for the quantitative measurement of the retinal thickness were from the same eyes used in the H&E staining. Quantification of the retinal thickness was performed in 4 *μ*m thick sagittal sections of the eyeball. Three intact sections were evaluated at 3 sites 52 *μ*m adjacent to the optic nerve head and along the periphery, each separated by a distance of 20 *μ*m. In each selected section under 40x objective, the retinal thickness at three sites per section was measured by ImageJ. The thickness was measured across the different retinal layers including the nerve fiber layer (NFL), ganglion cell layer (GCL), inner plexiform layer (IPL), inner nuclear layer (INL), outer plexiform layer (OPL), and outer nuclear layer (ONL).

The sections for the quantitative counting of the ganglion cells were the same used in retinal thickness assay. Three intact sections were evaluated in each eye, and ganglion cells were counted under 20x objective. The linear length of retina measured was about 600 *μ*m.

### 2.9. Statistical Analysis

Data were presented as mean ± SD and percentage. Data obtained in diverse groups were assessed with an unpaired Student *t*-test using SPSS 16.0 (SPSS Inc., Armonk, NY). One-way ANOVA was carried out to assess within-group differences of immunofluorescence intensity. A probability value of *p* < 0.05 was considered significant.

## 3. Results

### 3.1. Neuronal and Vascular Changes during Diabetes Progression

#### 3.1.1. Animals: Physical and Clinical Observations

The body weight of diabetic rats in the DM3w group was significantly lower than that in the NC group (325 ± 16 versus 294 ± 44; *p* < 0.01). The blood glucose level was maintained at a high level after diabetes induction.

#### 3.1.2. H&E Staining

**Figure 1:**
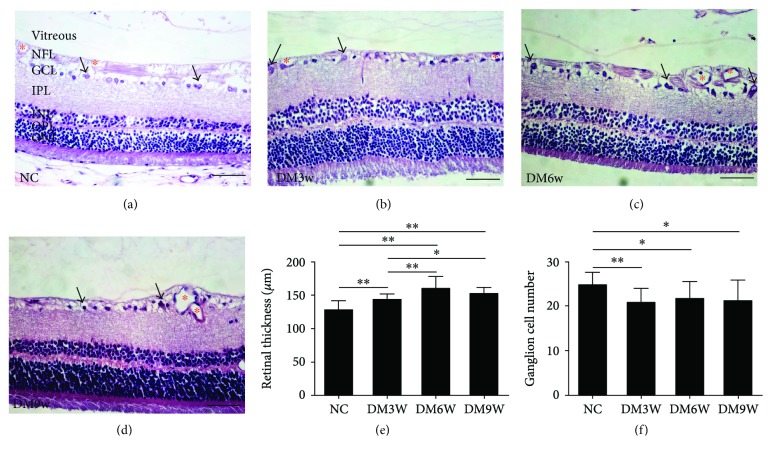
Neuronal and vascular changes in the early stage of diabetes. (a–d) H&E staining of the retina in the NC, DM3w, DM6w, and DM9w groups. Black arrow indicates neuronal changes in which the ganglion cells appeared to be enlarged at the DM3w group but were condensed at the DM6w and DM9w groups. Blood vessels were dilated in diabetic retina notably at the DM6w and DM9w groups (asterisk). (e, f) Quantification of retinal thickness and ganglion cell number in the NC, DM3w, DM6w, and DM9w groups. Bar = 50 *μ*m. ^∗^ *p* < 0.05 and ^∗∗^ *p* < 0.01.

H&E staining showed that the retina of diabetic rats did not present obvious structural changes at 3w after diabetes induction. Nevertheless, retinal thickness was increased in the DM3w, DM6w, and DM9w groups. When compared with the DM3w group, the retinal thickness was significantly higher in the DM6w and DM9w groups (Figure 1(e)). The results of enumeration of ganglion cells showed that the cell number was decreased in the DM3w, DM6w, and DM9w groups (Figure 1(f)).

Compared with the NC and DM3w groups, the retina in the DM6w group exhibited edema and retinovascular dilation, while neural and endothelial cells remained relatively normal (Figures 1(a)–1(c)). In the DM9w group, the retinal vasculature underwent more pathological changes such as vitreous degeneration, thickening of vascular wall, and luminal stenosis. In addition, the nerve fiber layer (NFL) of the retina became thinner (Figure 1(d)).

#### 3.1.3. Double Immunofluorescence Labeling

**Figure 2:**
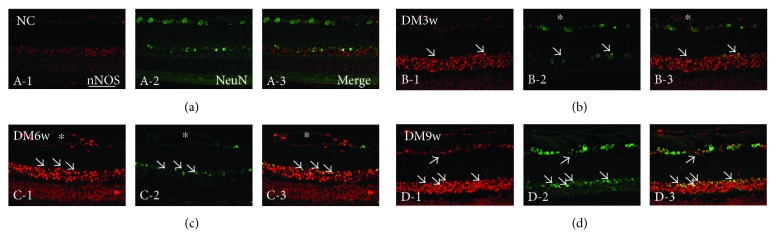
Representative photomicrographs of nNOS (red) and NeuN (green) double staining of the retina in the NC and DM6w groups. nNOS immunoreactivity was localized in NeuN-labeled neurons (single arrow) and blood vessels (asterisk), and it was relatively higher in the DM6w group (B-1) than in the NC group (A-1). Moreover, coexpression of nNOS and NeuN was apparent in the DM6w group (B-3) compared to the NC group (A-3). Blood vessels appeared to be dilated in diabetic retina at the DM6w group (asterisk). Note the enhanced nNOS staining and smaller size of NeuN-positive neurons at the DM6w group compared with the control group. Bar = 50 *μ*m.

In the control rats, a moderate nNOS labeling was detected in different layers of the retina (Figure 2). nNOS expressing cells were identified to be the neurons as they coexpressed NeuN (Figure 2(a), A-1). In the DM3w, DM6w, and DM9w groups, the incidence of nNOS expressing neurons was increased and nNOS immunofluorescence was more intense in comparison with the matching control (Figures 2(a) and 2(b), B-1).

**Figure 3:**
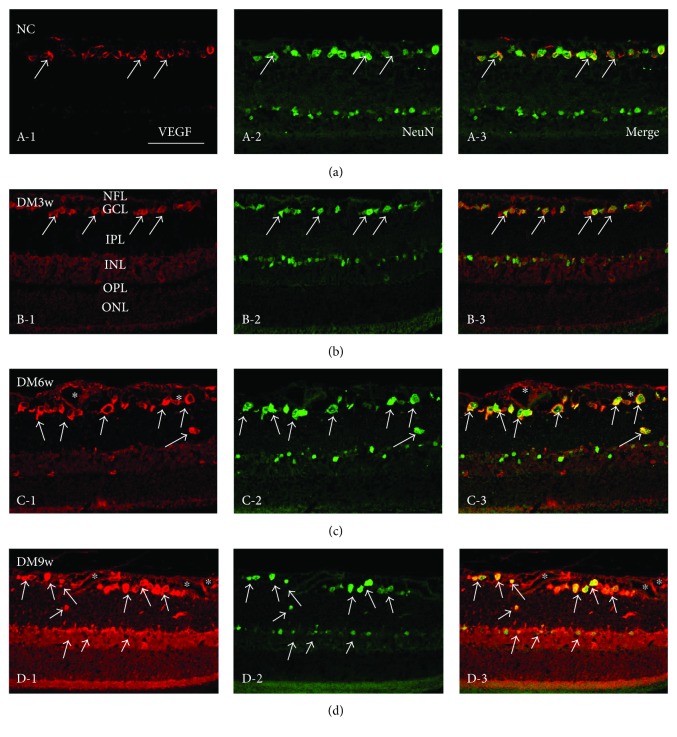
Representative photomicrographs of VEGF (red) and NeuN (green) double staining of the retina in the NC, DM3w, DM6w, and DM9w groups. VEGF immunoreactivity was localized in NeuN-labeled neurons (single arrow) and blood vessels (asterisk). VEGF staining appeared to be enhanced with the progress of diabetes (a, b, c, D-1), while the size of NeuN-labeled neurons became smaller (a, b, c, D-2). Bar = 50 *μ*m.

In the control rats, VEGF labeling was detected in the NFL, ganglion cell layer (GCL), and inner plexiform layer (IPL) (Figure 3(a), A-1). In the DM3w, DM6w, and DM9w groups, VEGF expression was conspicuously augmented in various retinal layers. VEGF immunoreactivity was most intense in the ganglion cells, whose identification was confirmed by double labeling with anti-VEGF and anti-NeuN (Figures 3(a)–3(d), D-3). Some neurons in the inner nuclear layer (INL) also exhibited colocalization of VEGF and NeuN at the DM6w and DM9w groups (Figures 3(c) and 3(d), D-3).

**Figure 4:**
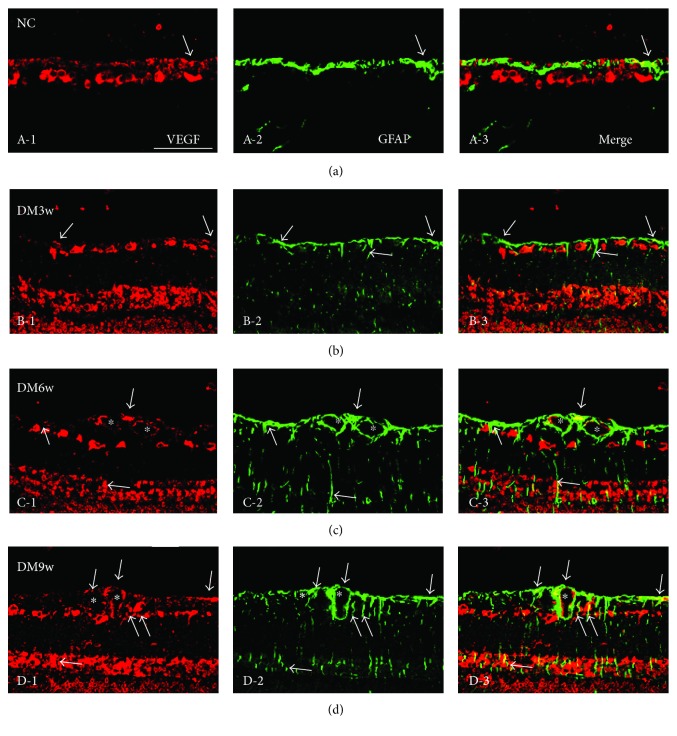
Representative photomicrographs of VEGF (red) and GFAP (green) double staining of the retina in the NC, DM3w, DM6w, and DM9w groups. VEGF immunoreactivity was localized in GFAP-labeled astrocytes (single arrow), blood vessels (asterisk), and Müller cells with their processes spanning across different retinal layers (double arrows). Bar = 50 *μ*m.

In the normal control rats, astrocytes were confined to the NFL and GCL and emitted GFAP immunofluorescence (Figure 4(a), A-2). Colocalization of VEGF labeling with GFAP was evident, but it was only detected in the GCL (Figure 4(a), A-3). In the DM3w, DM6w, and DM9w groups, the incidence of VEGF expressing astrocytes was increased (Figures 4(b)–4(d), D-3), and their long extending and hypertrophic processes were intensely stained with GFAP (Figures 4(b)–4(d), D-2). Müller cell processes in palisade spanned the different retinal layers (Figures 4(c) and 4(d), D-2); such a configuration was especially pronounced at the DM9w group. Enhanced VEGF expression was most conspicuous at 9w (Figure 4(d), D-1), in comparison with other time points (Figures 4(b) and 4(c), C-1). Except for some vascular profiles, GFAP-positive astrocytes in NFL were colocalized with VEGF immunofluorescence (Figures 4(c) and 4(d), D-3). Both VEGF and GFAP immunoreactivity in astrocytes or associated with the blood vessels were markedly enhanced after disease exposure (Figures 4(c) and 4(d), D-3).

**Figure 5:**
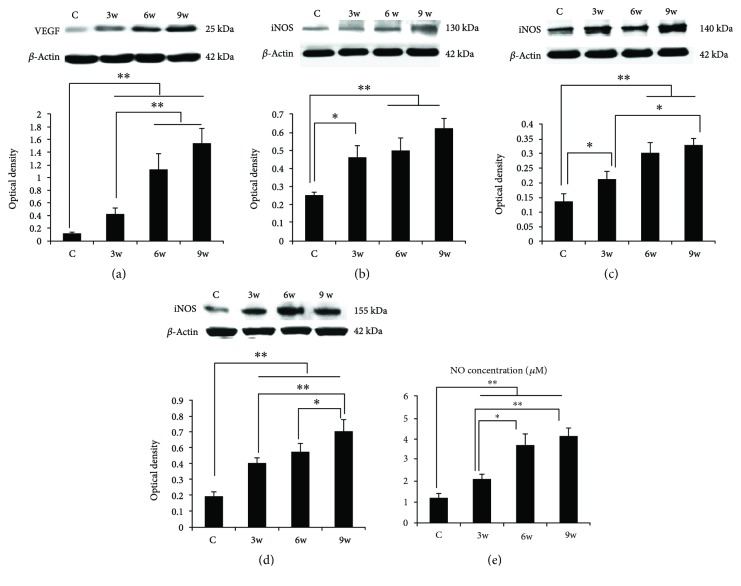
Western blot analysis of VEGF (a), iNOS (b), eNOS (c), and nNOS (d) protein expression levels in the retina in the NC, DM3w, DM6w, and DM9w groups, including the immunoreactive bands of VEGF (21 kDa), iNOS (130 kDa), eNOS (140 kDa), nNOS (155 kDa), and *β*-actin (42 kDa). Bar graphs representing optical density (mean ± SD). The expression levels were all increased after diabetes induction. (e) NO concentration in the NC, DM3w, DM6w, and DM9w groups. NO concentration was significantly increased in the retina at 3w, 6w, and 9w after diabetes induction when compared with that of control rats. ^∗^ *p* < 0.05 and ^∗∗^ *p* < 0.01.

Western blot analysis showed that VEGF and NOS proteins were increased after diabetes induction **(**Figures 5(a)–5(d) **)**. Compared with the NC group, densitometry of the VEGF immunoreactive band showed a significant increase at the DM3w (*p* < 0.01), DM6w (*p* < 0.01), and DM9w groups (*p* < 0.01). VEGF expression level was significantly higher in the DM6w (*p* < 0.01) and DM 9w (*p* < 0.01) groups than that in the DM3w group. nNOS expression level was also increased at the DM3w (*p* < 0.01), DM6w (*p* < 0.01), and DM9w groups (*p* < 0.01). Among the three groups, protein expression was the highest in the DM9w group than that in the DM3w (*p* < 0.01) group and the DM6w group (*p* < 0.05). The expression level of iNOS was increased in the DM3w (*p* < 0.05), DM6w (*p* < 0.01), and DM9w groups (*p* < 0.01), while the difference between the DM6w and DM9w groups was insignificant. The expression changes in pattern of eNOS followed that of iNOS, except that eNOS expression in the DM9w group was significantly higher than that in the DM3w group (*p* < 0.05).

Measurement of NO production showed that it was increased with the progression of diabetes (Figure 5(e) **)**. It was significantly higher in the DM3w (*p* < 0.01), DM6w (*p* < 0.01), and DM9w groups (*p* < 0.01). Compared with the DM3w group, NO level was increased significantly in the DM6w (*p* < 0.05) and DM9w groups (*p* < 0.01).

### 3.2. Bevacizumab Treatment Ameliorated Retinal Pathological Changes in the Early Stage of Diabetes

#### 3.2.1. H&E Staining

**Figure 6:**
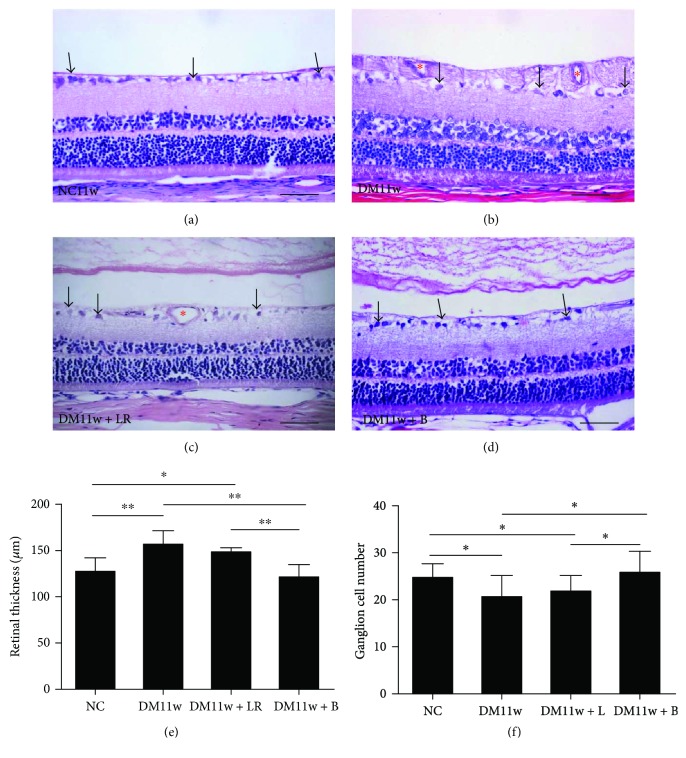
H&E staining of the retina in the NC11w, DM11w, DM11w + LR, and DM11w + B groups. (a–d) Note the cellular morphological changes (single arrow) in the DM11w and DM11w + LR groups compared with the NC11w and DM11w + B groups. Blood vessels were dilated in diabetic retina notably at the DM11w and DM11w + LR (asterisk). (e, f) Quantification of retinal thickness and ganglion cell number each group. Bar = 50 *μ*m. ^∗^ *p* < 0.05 and ^∗∗^ *p* < 0.01.

To study the effect and mechanism of anti-VEGF treatment on the early stage of DR, we adopted intravitreous injection of bevacizumab at 6 weeks after diabetes induction and evaluated its effect at 11w after diabetes induction. H&E staining showed that the retina from the DM11w and DM11w + LR groups exhibited structural alterations, such as decrease in ganglion cells, vascular dilation in both the inner limiting membrane (ILM) and NFL, and thickening of inner plexiform layer (Figures 6(a)–6(c)). By contrast, the retina in the diabetic rats from the DM11w + B group did not show significant changes; rather, it appeared to be comparable to the NC11w group (Figure 6(d)). When compared with the NC and DM11w + B groups, the retinal thickness was increased in the DM11w and DM11w + LR groups (Figure 6(e)). Furthermore, quantification of ganglion cells showed that the ganglion cell number was decreased in the DM11w and DM11w + LR groups (Figure 6(f)).

#### 3.2.2. Double Immunofluorescence Labeling

**Figure 7:**
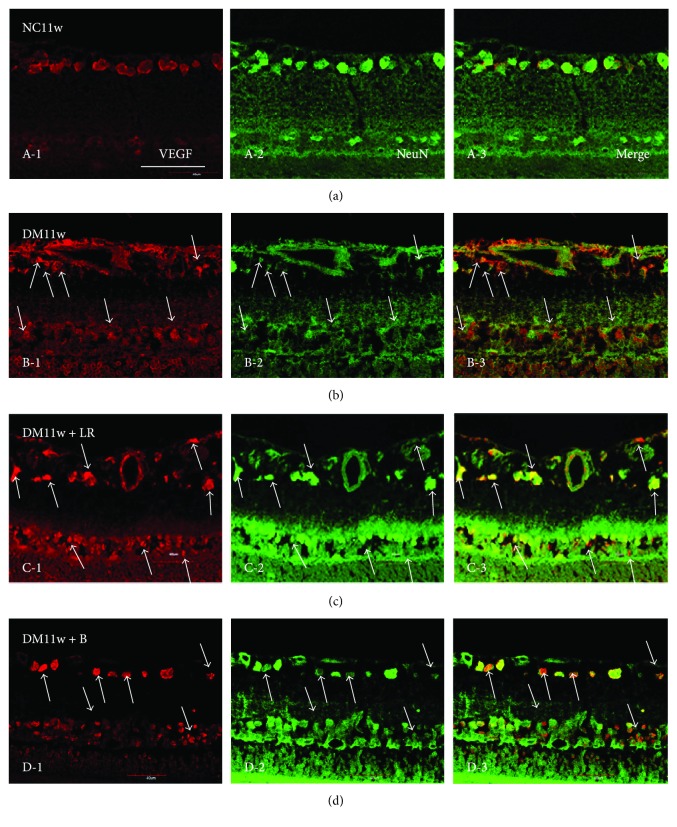
Representative photomicrographs of VEGF (red) and NeuN (green) double staining of the retina in the NC11w, DM11w, DM11w + LR, and DM11w + B groups. VEGF immunoreactivity was localized in NeuN-labeled neurons (single arrow) and blood vessels (asterisk). (a, b, c, D-1) The expression of VEGF was notably enhanced in the DM11w and DM11w + LR groups compared with the NC11w and DM11w + B groups. (a, b, c, D-2) Moreover, NeuN-labeled neurons appeared to be condensed and exhibit morphological changes in the DM11w and DM11w + LR groups, in comparison with the NC11w and DM11w + B groups. Bar = 50 *μ*m.

**Figure 8 fig8:**
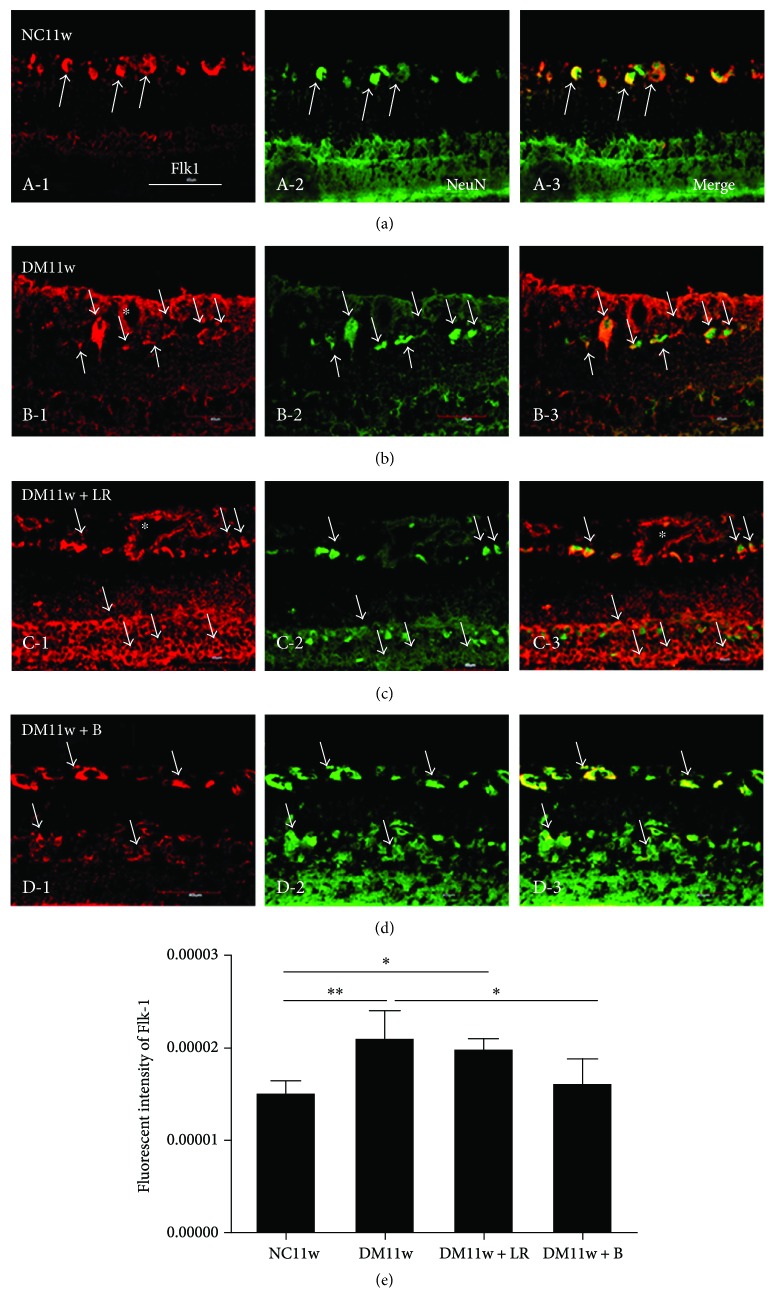
Representative photomicrographs of Flk-1 (red) and NeuN (green) double staining of the retina in the NC11w, DM11w, DM11w + LR, and DM11w + B groups. (a, b, c, D-1) Flk-1 immunoreactivity appeared to maintain its structural integrity and to be lower in the NC11w and NC11w + B groups (single arrow). (e) Semiquantification of Flk-1 by immunofluorescence. Note the dilated blood vessels in diabetic retina at the DM11w and DM11w + LR (asterisk). Bar = 50 *μ*m. ^∗^ *p* < 0.05 and ^∗∗^ *p* < 0.01.

**Figure 9 fig9:**
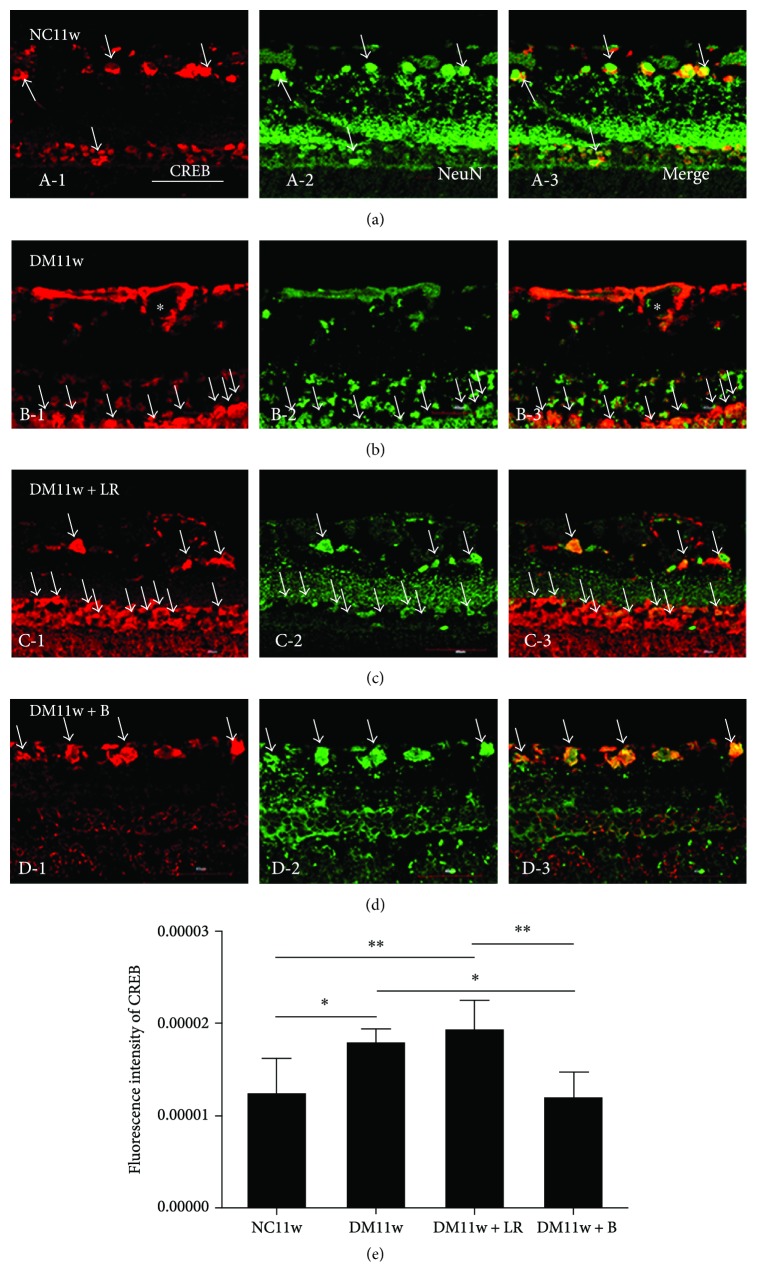
Representative photomicrographs of CREB (red) and NeuN (green) double staining of the retina in the NC11w, DM11w, DM11w + LR, and DM11w + B groups. CREB immunoreactivity is localized in NeuN-labeled neurons (single arrow) and blood vessels (asterisk). (b, C-1) In the DM11w and DM11w + LR groups, CREB expression was notably increased and even could be found in IPL. (D-1) However, CREB almost expressed only in GCL in the DM11w + B group. (e) Semiquantification of CREB by immunofluorescence. Bar = 50 *μ*m. ^∗^ *p* < 0.05 and ^∗∗^ *p* < 0.01.

**Figure 10 fig10:**
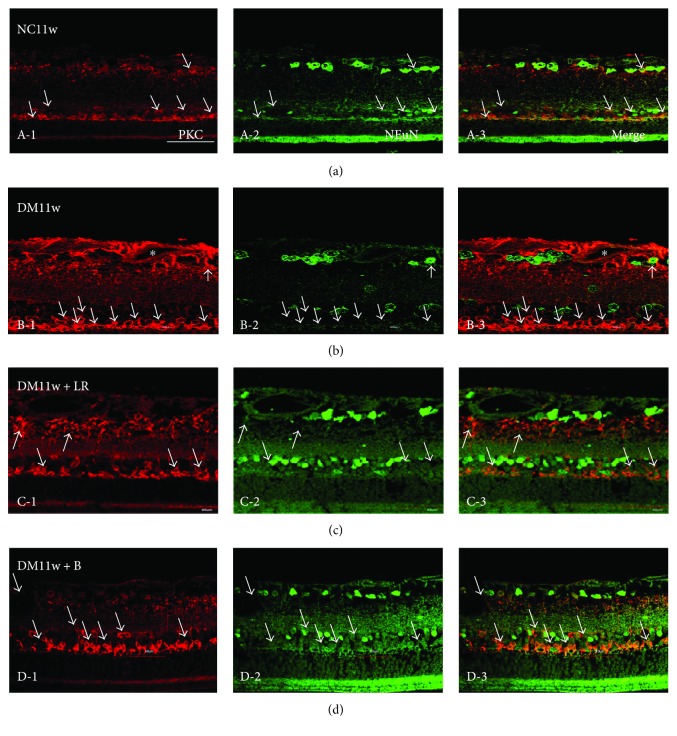
Representative photomicrographs of PKC (red) and NeuN (green) double staining of the retina in the NC11w, DM11w, DM11w + LR, and DM11w + B groups. PKC immunoreactivity was localized in NeuN-labeled neurons (single arrow) and blood vessels (asterisk). Bar = 50 *μ*m.

**Figure 11:**
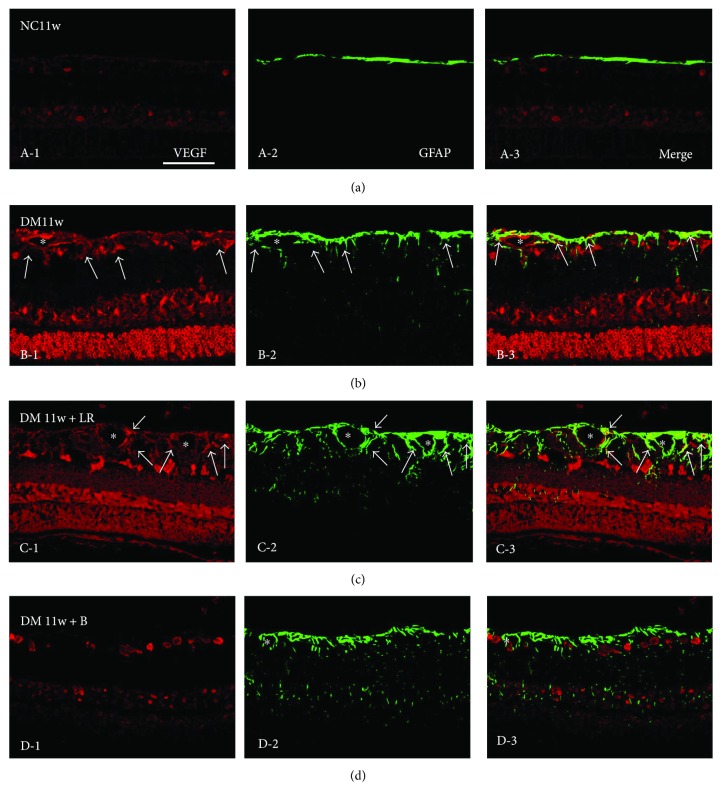
Representative photomicrographs of VEGF (red) and GFAP (green) double staining of the retina in the NC11w, DM11w, DM11w + LR, and DM11w + B groups. VEGF immunoreactivity was localized in GFAP-labeled astrocytes (single arrow) and blood vessels (asterisk). (b, C-2) GFAP expression appeared to be enhanced in the DM11w and DM11w + LR, consistent with the expression of VEGF in these two groups. (A-1, 2, D-1, 2) By contrast, both VEGF and GFAP expressions were relatively lower in the NC11w and DM11w + B groups. Note the dilated blood vessels in diabetic retina at the DM11w + LR group (asterisk). Bar = 50 *μ*m.

Immunostaining showed that VEGF, Flk-1, CREB, and PKC proteins were mainly expressed in GCL, INL, and outer nuclear layer (ONL). The protein expression of these proteins was higher in the DM11w and DM11w + LR groups than in the NC11w and DM11w + B groups. However, Flk-1 and CREB expression was mainly localized within GCL in the retina of normal rats, while PKC expression was spread into INL. In the DM11w + B group, CREB expression was reduced in GCL, while Flk-1 and PKC expression remained in INL and ONL (Figures 7 [Fig fig8] [Fig fig9] [Fig fig10]–11).

Immunostaining results showed that the DM11w and DM11w + LR groups had more VEGF-positive cells than the NC11w group localized mainly in GCL and ONL. Furthermore, in the DM11w + LR group, the blood vessels in the ILM and NFL appeared to be dilated. VEGF expression was localized in the endothelial cells. After treatment with bevacizumab, VEGF expression was evidently decreased. Along with this, the structural integrity of blood vessels was relatively intact in the DM11w + B (Figure 7).

In the control rats, Flk-1 immunofluorescence was evident in NFL and GCL and relatively weak in INL (Figure 8(a)). Cells expressing Flk-1 were colocalized with neurons expressing NeuN (Figures 8(a)–8(d), D-3). Compared with the NC11w group, Flk-1 expression was significantly increased in the DM11w group and the DM11w + LR group (Figures 8(b), 8(c), C-1, and 8(e)). In these two groups, Flk-1/NeuN coexpressing cells were distributed in GCL and IPL. And Flk-1 expression was significantly decreased in the DM11w + B group compared with the DM11w group (Figures 8(d), D-3 and 8(e)).

CREB immunofluorescence was evident in NFL, GCL, and ONL in control rats (Figure 9(a), A-1). In diabetes-induced rats, CREB expression was noticeably increased in ONL and coexpressed with neurons expressing NeuN (Figures 9(a)–9(d), D-3). After bevacizumab treatment, CREB immunofluorescence was limited to NFL and GCL (Figure 9(d), D-3). Compared with the control rats, CREB expression in all three diabetes groups overlapped with NeuN expression (Figures 9(b)–9(d), D-3). Semiquantification of its fluorescent intensity showed that CREB expression was significantly increased in the DM11w and DM11w + LR groups (Figure 9(e)).

Moderate PKC immunoreactivity was detected in ONL, GCL, and NFL in control rats (Figure 10(a), A-1). In diabetes-induced rats, PKC expression was markedly augmented; moreover, it was also expressed in neurons in INL (Figures 10(b)–10(d), D-3). After bevacizumab treatment, PKC expression was noticeably reduced that was localized mainly in ONL (Figure 10(d), D-3).

In the control rats, astrocytes confined to the NFL and ganglion cell layer (GCL) exhibited GFAP immunofluorescence (Figure 11(a), A-2). A moderate VEGF labeling was detected in the NFL, GCL, INL, and ONL (Figure 11(a), A-1). Colocalization of GFAP and VEGF was very minimal that was detected in the GCL only (Figure 11(a), A-3). In the DM11w and DM11w + LR groups, Müller cells intensely stained for GFAP and whose cell bodies residing in the GCL were hypertrophied (Figures 11(b), B-2 and 11(c), C-2). The cells exhibited long extending processes (Figures 11(b), B-2 and 11(c), C-2), but they appeared attenuated at the DM11w + B group (Figure 11(d), D-2). Double labeling with anti-VEGF and anti-GFAP had revealed that the VEGF-positive cells were astrocytes (Figures 11(b)–11(d), D-3).

**Figure 12:**
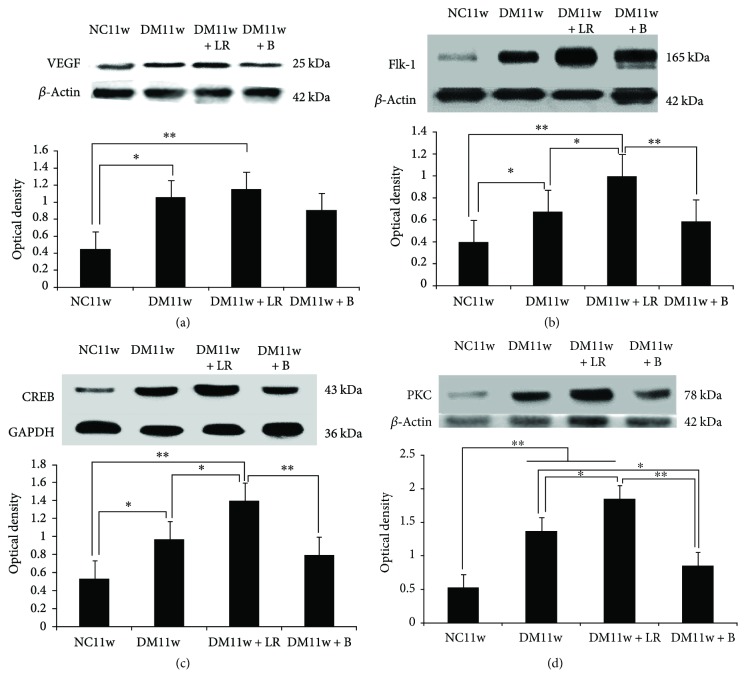
(a) Western blot analysis showed the expression of VEFG, Flk-1, CREB, and PKC in the NC11w, DM11w, DM11w + LR, and DM11w + B groups, including the immunoreactive bands of VEGF (21 kDa), iNOS (130 kDa), eNOS (140 kDa), nNOS (155 kDa), and *β*-actin (42 kDa). Bar graph showed the semiquantitative data of Western blot analysis of VEGF (a), Flk-1 (b), CREB (c), and PKC (d). (a) VEGF showed an evident increase in the DM11w and DM11w + LR groups compared with the NC11w group. (b–d) Flk-1, CREB, and PKC showed significant increase in the DM11w and DM11w + LR groups compared with the NC11w group, which was attenuated in the DM11w + B group. ^∗^ *p* < 0.05 and ^∗∗^ *p* < 0.01.

Western blot analysis showed that VEGF protein expression in the DM11w (*p* < 0.05) and DM11w + LR groups (*p* < 0.01) was significantly higher than that in the NC11w group. In the DM11w + B group, VEGF protein expression level was higher than that in the NC11w group, but it was lower than that in the DM11w and DM11w + LR groups; however, the difference was not significant (Figure 12(a)).

Flk-1 protein expression of the retina in the DM11w (*p* < 0.05) and DM11w + LR groups (*p* < 0.01) was significantly higher than that in the NC11w group. In the DM11w + B group, Flk-1 protein expression was significantly lower than that in the DM11w + LR group (*p* < 0.01) (Figure 12(b)).

CREB protein expression of retina in the DM11w (*p* < 0.05) and DM11w + LR groups (*p* < 0.01) was significantly higher than that in the NC11w group. In the DM11w + B group (*p* < 0.01) and the DM11w group (*p* < 0.05), it was significantly lower than that in the DM11w + LR group (Figure 12(c)).

PKC protein expression of the retina in the DM11w (*p* < 0.01) and DM11w + LR groups (*p* < 0.01) was significantly higher than that in the NC11w group. In the DM11w + B group, it was higher than that in the NC11w (*p* < 0.05) and DM11w + LR groups (*p* < 0.01) (Figure 12(d)).

## 4. Discussion

The present results have shown that diabetes could result in progressive neuronal and vascular damage in the retina from 3 to 9 weeks after diabetes induction. We have shown that anti-VEGF treatment using bevacizumab could not only prevent retinal pathological changes but also exert beneficial effects on the underlying molecular mechanism, mainly through limiting an undesirable increase in VEGF. In this connection and although other experimental studies have shown the protective effect of anti-VEGF treatment [13, 19–22], the changes of neural and vascular elements of retinopathy pathogenesis after VEGF inhibition have remained uncertain. Therefore, clarifying how diabetes might affect the neural retina is crucial to the development of new therapeutic agents. Compared with previous studies, here, we have examined both neuronal and vascular changes in DR. In the latter, and importantly, we showed here that VEGF maybe involved in the neuronal damage through NOS. To ascertain this, we have adopted the application of bevacizumab as a preventive strategy for DR. Thus, in this study, we have closely monitored the diabetes-induced pathological changes and intervened at the early phase of diabetes with bevacizumab. Additionally, we measured the VEGF and NOS protein expression before and after bevacizumab treatment and explored their mutual role in anti-VEGF treatment. Finally, we tested changes of some relevant factors and their possible involvement in DR.

### 4.1. VEGF, NOS, and Inflammation

VEGF has long been regarded as an important factor of DR [23], and its relationship with nitric oxide has been extensively investigated. VEGF could activate eNOS in vascular endothelial cells, which could then change the vascular permeability and stimulate angiogenesis [24]. Flk-1, which is a vascular endothelial growth factor receptor 2 (VEGFR2), plays a key role in these processes, activating eNOS with its autophosphorylation [25], and topical application of a novel VEGFR2/Src kinase inhibitor could suppress retinal vascular permeability in mice and rabbits [19]. In turn, NO could induce VEGF synthesis [26]. Previous study found that VEGF and eNOS were increased in the retina of diabetic rats and VEGF inhibitor could potently suppress the increase of eNOS and thus reduce retinal inflammation [27]. In addition to eNOS, iNOS was critical in the early pathogenesis of vascular lesions of DR. Using iNOS-deficient mice, it was reported that mice lacking iNOS were protected from the diabetes-induced degeneration of retinal capillaries [28]. Moreover, the upregulation of iNOS, not eNOS and nNOS, accounts for the compromised retinal function in DR through increasing NO levels [5]. Therefore, iNOS has been identified as one of the most important culprits in vascular changes associated with the early stages of DR [29]. Our previous studies have found that VEGF and NOS protein expression was increased under hypoxia condition both in the brain [30] and the retina [31] and NOS inhibitors effectively reduced the vascular leakage.

VEGF is reported to be chemotactic for monocytes *in vitro* [32] and to significantly enhance recruitment of monocytes *in vivo* [33]. Furthermore, VEGF is a potent enhancer of vascular permeability [34], and thus resulting in increased leukocyte recruitment [35]. The effect of VEGF on inflammation is thought to be important for angiogenesis [36, 37].

In this study, we found that VEGF protein expression was elevated with the progression of diabetes from 3 to 9 weeks. VEGF protein was mainly expressed in the blood vessels and cells in the ganglion cell layer and ONL. Meanwhile, the protein level of iNOS, eNOS, and nNOS and production of NO was increased. Remarkably, in DR rats given treatment with bevacizumab, the preservation and integrity of cellular and vascular structure appeared to correlate with decrease in VEGF and NOS protein expression. It stands to the reason therefore that NOS proteins are important factors regulated by VEGF which can cause neuronal damage in the retina of diabetic rats.

### 4.2. Bevacizumab Treatment

Many studies supported that the inhibition of VEGF bioactivity could be an effective treatment for the early diabetic retinopathy [27, 38]. Bevacizumab has been approved for the treatment of metastatic colorectal cancer [39] and become a current off-label treatment by ophthalmologists for neovascular age-related macular disease [3]. Several clinical trials have confirmed the effect of bevacizumab on ocular disease, especially on proliferative diabetic retinopathy [10, 15, 16].

Here, we present evidence supporting that bevacizumab can maintain the structural integrity in the diabetic retina. It has been reported that widened retinal venular caliber is independently associated with prevalence and progression of diabetic retinopathy and predicts risk of proliferative retinopathy [40]; we postulate that bevacizumab treatment as shown in this study has a preventive effect on DR.

### 4.3. The Underlying Mechanism of Anti-VEGF Therapy

To gain a better understanding of the underlying molecular mechanism that may be involved in bevacizumab treatment, we have explored for some crucial factors. To this end, the expression levels of PKC, VEGF, Flk-1, and CREB proteins were significantly increased in the diabetic rats; more strikingly, the expression was suppressed after anti-VEGF treatment. Concurrent to this, the neuronal and vascular changes in DR were restored showing histological features that were comparable to those of the control rats.

We next evaluated the protein expression of PKC because diacylglycerol- (DAG-) protein kinase (PK) C pathway is a very important pathway in cellular signaling induced by diabetes [41, 42]. During the pathogenesis of DR, PKC, activated by DAG, causes retinal vascular dysfunction by changing NO and VEGF enzyme activities in both endothelial cells and pericytes [43]. Moreover, it has been documented that inhibition of PKC could protect ECs from the damage of high glucose [29]. In the present study, we found that PKC protein expression was increased in diabetic rats and was significantly higher than that in normal rats. As a corollary, we hypothesized that bevacizumab treatment may exert its effects through DAG-PKC pathway.

Flk-1 (VEGFR2) is a decisive factor in the induction of angiogenesis and PKC activation [44]. Previous study has found that VEGF/Flk-1 signaling could phosphorylate CREB and exert protective effect on neurons and endothelial cells. We also found that anti-VEGF treatment decreased the expression of CREB [45]. Though CREB may be beneficial for hypoxic damage, it may contribute to the pathogenesis of diabetic retinopathy. Previous study has found that blocking CREB will affect the high glucose-induced VEGF expression, thus preventing activation of retinal Müller cells and enhanced angiogenesis [46]. In view of the above, it is suggested that anti-VEGF therapy may attenuate diabetic retinopathy through inhibiting NO production as well as affecting PKC and CREB expression.

### 4.4. Some Considerations about Anti-VEGF Therapy

Despite all these existing positive results, it needs to be cautioned that there remain some issues concerning the use of anti-VEGF therapy [44]. For example, the half-life of bevacizumab is short. To elicit and retain the required therapeutic response, repeated maintenance doses are required. In our study, we also found that intravitreal injection of sodium lactate Ringer's led to more severe retinovascular changes and higher expression of Flk-1, CREB, and PKC compared with the DM11w group. It is also noteworthy that the cognitive function of diabetic rats was not aggravated by an intravitreal injection of bevacizumab (data not shown). Although there are reports of the adverse effects following anti-VEGF therapy, such as tractional retinal detachment, macular hole formation, and foveal avascular zone enlargement [38], these have not been noted in our DR model; moreover, clinical trials on the use of intravitreal anti-VEGF therapy for treatment of diabetic retinopathy generally show low rates of side-effects [14, 16, 47].

All in all, our study still has some limitations. Firstly, for clinical relevance, we should have considered applying glycemic control to diabetic rats. However, our study focused mainly on the early pathogenesis of the diabetic retinopathy and studied its progression without interference. In addition, we found that retinal thickening and RGC loss occurred much earlier than in other studies [48]. We attribute the difference to the factor of insulin therapy. We postulated that diabetic pathological changes progressed rapidly without insulin intervention. Our previous study has supported this postulation, finding that diabetes caused arteries narrowing in the circle of Willis under synchrotron radiation angiography as early as 2 weeks [18].

## Supplementary Materials

Supplementary Materials Figure S1: hematoxylin and eosin stain of sham rat eyeball for measurement of retinal thickness. At least three intact 20 *μ*m sections were taken from the periphery (#), inclusive of the retina adjacent to the optic nerve head (^∗^). Bar = 1 mm.
