# Supplementary material for: Intravitreal Bevacizumab Injection Attenuates Diabetic Retinopathy in Adult Rats with Experimentally Induced Diabetes in the Early Stage
Source: J Diabetes Res. 2018 May 9;2018:9216791. doi: 10.1155/2018/9216791 (PMC5994287; doi:10.1155/2018/9216791)
Supplement: Supplementary Materials — Figure S1: hematoxylin and eosin stain of sham rat eyeball for measurement of retinal thickness. At least three intact 20 μm sections were taken from the periphery (#), inclusive of the retina adjacent to the optic nerve head (∗). Bar = 1 mm. [file 9216791.f1.docx]

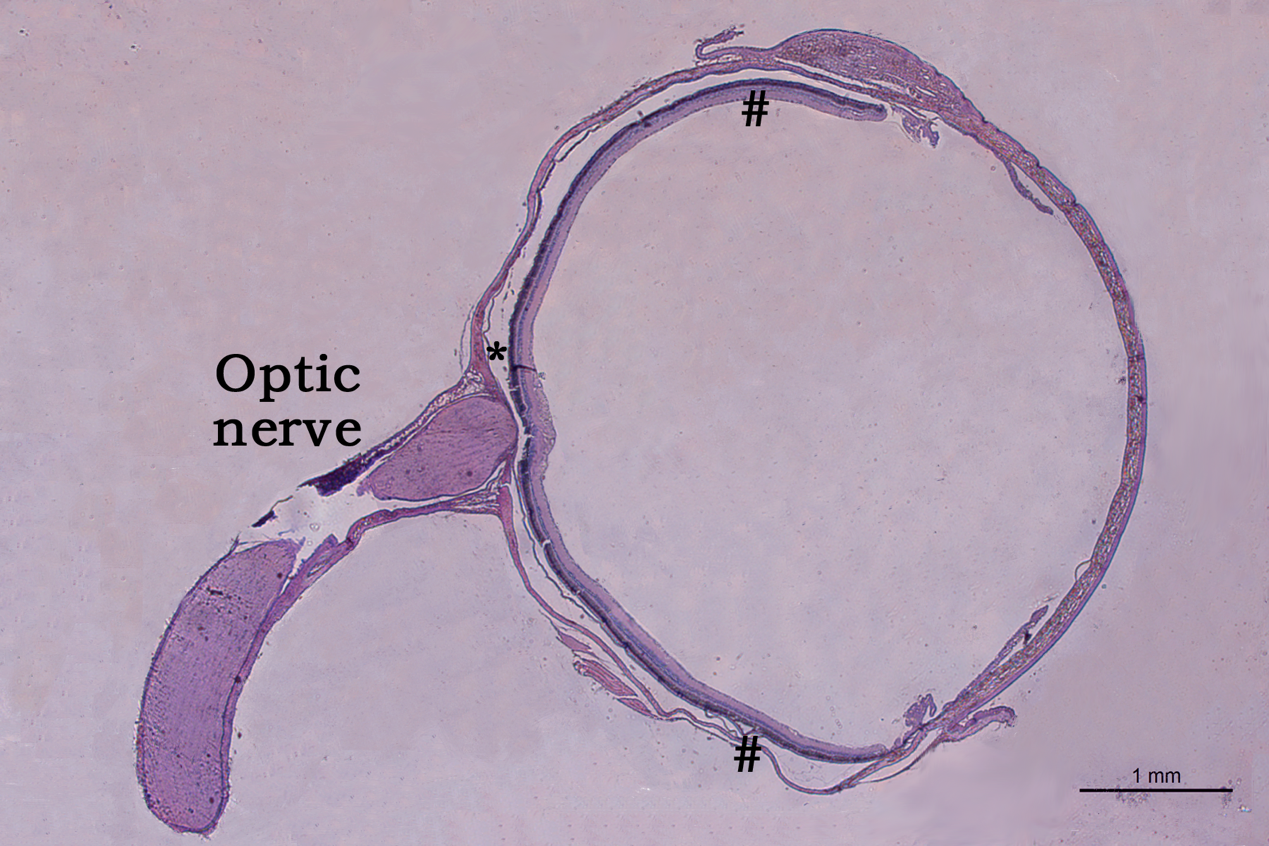


Figure S1. Hematoxylin and eosin stain of sham rat eyeball for measurement of retinal thickness. At least three intact 20-μm sections were taken from the periphery (#), inclusive of the retina adjacent to the optic nerve head (*).
